# Influence of Different Prebiotics on Viability of *Lactobacillus casei*, *Lactobacillus plantarum* and *Lactobacillus rhamnosus* Encapsulated in Alginate Microcapsules

**DOI:** 10.3390/foods10040710

**Published:** 2021-03-26

**Authors:** Liliana Luca, Mircea Oroian

**Affiliations:** Faculty of Food Engineering, Stefan cel Mare University of Suceava Romania, 720229 Suceava, Romania; liliana.luca@usm.ro

**Keywords:** alginate beads, encapsulation, *Lactobacillus* spp., viability

## Abstract

As the production and maintenance of a sufficient number of microencapsulated probiotics is still a test for the food industry, the present study addressed the testing of three prebiotics: chicory inulin, soluble potato starch, oligofructose and a control carbon source, namely glucose, as a component part of the encapsulation matrix. Using the extrusion encapsulation technique, it was possible to obtain microcapsules whose matrix composition and dimensions correspond to the requirements of the food industry. The microcapsules obtained showed significantly different physicochemical properties, with different survival rates during processing, storage and in simulated gastrointestinal conditions. The encapsulation efficiency was very high in relation to the dimensions of the microcapsules and the technique used (between 87.00–88.19%). The microcapsules obtained offered a very good viability (between 8.30 ± 0.00–9.00 ± 0, 02 log10 cfu/g) during the 30 days of storage at 2–8 degrees and also in the simulated gastrointestinal conditions (between 7.98–8.22 log10 cfu/g). After 30 days, the lowest viability was registered in the microcapsules with glucose 6.78 ± 0.15 log10 cfu/g. It was found that after 4 h of action of gastrointestinal juices on the microcapsules stored for 30 days, cell viability falls within the limits recommended by the Food and Agriculture Organization of the United Nations (FAO) (10^6^–10^7^ CFU/mL or g of food. This study demonstrated that using prebiotic encapsulation matrix increases cell viability and protection and that the extrusion encapsulation method can be used in the production of probiotic microcapsules for the food industry.

## 1. Introduction

In contemporary scientific literature, a growing interest has been observed in the importance of the intestinal microbiota in human health. There is much scientific evidence proving that probiotics can protect the human body against numerous diseases from infections to degenerative diseases and also psychological damage [1,2,3]. The probiotics success in health promotion is highly dependent on both cell viability and on the number reaching the intestinal tract. According to Food and Agriculture Organization of the United Nations (FAO) and World Health Organization (WHO) [4] “The content of live cells in the portion of the food recommended for daily consumption should be 10^9^ CFU/daily portion until the end of the product shelf-life, at the specified storage conditions, with uncertainty of 0.5 log”. It is important to note that the minimum number of live probiotic cells recommended to provide benefits to the host in the intestine is 10^6^–10^7^ CFU/mL or g of food. It has been found that probiotic cultures in many foods are no longer, at the end of the storage period, in the optimal number to support the health of the consumer [5,6,7,8,9,10].

It is known that these micro-organisms are sensitive to several factors (heat, environments with high acidity, the presence of oxygen, humidity, etc.) that can reduce their viability. This loss of cell viability practically decreases the effectiveness of the food administered. Both the technologies and the food matrix must aim to protect microorganisms against external stressors [6]. Incorporating these probiotics into microcapsules is an emerging method to maintain a high cell survival rate during food processing, storage and digestion, as well as an opportunity to control the release of these cells into the intestinal tract [11]. Most of these technologies are based on immobilizing bacteria in a polymeric matrix. Several materials were used for this matrix such as polysaccharides (alginate, chitosan, vegetable gums, k-carrageenan, cellulose acetate phthalate, etc.), proteins (gelatine, milk proteins) and fats [12,13,14].

Today, the most used methods for encapsulating probiotics are extrusion, freeze-drying, spray-drying, emulsification or phase separation [11], each of them with advantages and disadvantages [11,13,15].

One of the most used polymers as an encapsulation material is sodium alginate. This biopolymer forms a low cost non-toxic and biocompatible matrix that can offer protection to bioactive substances and also to microorganisms. Although sodium alginate is suitable for encapsulation, its gel is porous and sensitive to extreme pH values, thus affecting the release and the protection of targeted compounds [16]. There are several ways to overcome this obstacle and improve the stability of microorganisms, for example, by adding a nutrient substrate such as various sources of carbon, inulin, galactooligosaccharides, pectin, oligofructose, raw oats, etc., in the composition of the capsule [17]. The study of probiotics led to the development of prebiotics, which, in addition to the carbon source used by cells in metabolic processes, is also a bio protector for them. At this moment, in the literature, the combination of probiotics: *Lactobacillus casei 431*, *L. plantarum* and *L. rhamnosus* (Bioprox RP80) presented in the present research has not been studied. These strains are already used in the dairy food industry but each separately.

The first L. CASEI 431^®^ probiotic strain has been associated with health benefits within several areas of health, including immune health, respiratory healthy and bowel function. Bioprox RP80 culture includes two strains that have many probiotic effects on health. This culture is used for fresh cheese, stirred yogurt, drink yogurts, set yogurts, sour cream, cottage cheese and tvorog [18,19,20]. The current study aimed to evaluate different combinations of sodium alginate and three prebiotics such as inulin, oligofructose, potato starch in maintaining high probiotic cell viability in microcapsules for 30 days at 4 degrees and in simulated digestive juices. For control, glucose was selected as the carbon source. It was studied which combination is the most effective and the optimal formula for improving the survival of lactobacilli strains (*Lactobacillus casei*, *L. plantarum*, *L. rhamnosus*). Therefore, the strains chosen as the probiotic model were exposed to different stress factors which can be encountered during the encapsulation process, during storage at 4 °C and also after ingestion. At the same time, this study investigated the influence of prebiotics used as a carbon source on the growth of *Lactobacillus* strain biomass, on the physicochemical properties and cell viability as a result of the microencapsulation and storage process.

## 2. Materials and Methods

### 2.1. Preparation of Probiotic Microorganisms

In this study, 3 strains of lactobacilli were used: Lactobacillus casei 431 (Christian Hansen) and a mixed culture of Lactobacillus plantarum and Lactobacillus rhamnosus: Bioprox RP80 (Enzymes & Derivates, Piatra Neamt, Romania). The probiotic strains were reactivated 3 times at 37 °C for 24 h. The first and second reactivation was done for each strain separately. The second and third reactivation was done on modified MRS broth culture media while the first was made on MRS broth according to the original recipe. Modified MRS was prepared according to previous studies [21]. In the third reactivation, all three probiotic strains were combined in equal proportions. A McFarland densitometer was used for this with a McFarland measuring range of 0.3–15.0 at wavelength λ = 565 ± 15 nm. After the third reactivation, a 12–16 h inoculum (depending on the carbon source used: 12 h for glucose, 14 h for inulin and oligofructose and 16 h for starch) was used for encapsulation.

The carbon source used in modified culture media was represented by probiotics as: soluble potato starch (STH) and d-(+)-Glucose monohydrate (G) from Sigma-Aldrich, Nurnberg, Germany, chicory inulin (INU)/Fibruline^®^DS2-98%/inulin (Cosucra, Pecq, Belgium), oligofructose (OLI/FOS)—Cosucra, Pecq, Belgium. In order to check the sterility conditions of the culture media throughout each experiment, uncultivated culture media of each type were used as a negative control.

### 2.2. Prebiotic Activity Testing/Prebiotic Consumption

Prebiotic activity was determined by-biomass growth and pH monitoring. In order to determine the growth of cellular biomass, it was necessary to prepare modified culture media. The modification of the original medium was done by replacing the carbon source (glucose = 20 g/L^−1^) with prebiotics: INU, STH, OLI in the same ratio. Two control media were used, one without glucose and another in which glucose was added. Homogenization of the culture media was done for 10 min at 25 °C using a heated magnetic stirrer (Heidolph, MR Hei-Tec, Schwabach, Germany). Then, the pH of the modified MRS broth was adjusted to a value of 6.2 with hydrochloric acid before sterilization. Sterilization was performed at 121 °C in an autoclave for 20 min. Next, 1 mL of mixed culture was added to 9 mL of modified MRS broth of each type. Biomass growth was monitored by inoculation once every two hours for 12, 14 h on MRS agar from cultures grown on modified MRS broth. For this, serial dilutions were performed, of which 1 mL was taken for inoculation. Inoculations were performed in triplicate by inoculation in MRS agar medium. The plates were incubated for 48–72 h at 37 °C under anaerobic conditions. Results were expressed in colony forming units per ml (cfu/mL).

### 2.3. Encapsulation Process

Extrusion technique used for encapsulation was following the procedure described by Darjani et al., Krasaekoopt et al., Peredo et al. [15,22,23,24]. Sterilized utensils and glassware were used. Distilled water (distilled water machine-A4000D Aquatron, Cole-Parmer Ltd. Wertheim, Germany) used for the preparation of all solutions was sterilized by autoclaving (121 °C for 20 min-ICANCLAVE K [CLASS I] Ningbo Ican Machines Co., Quart de Poblet, Spain). The encapsulation process was modified and adapted to the needs of the present study. To obtain the capsule matrix, the following materials were used: sodium alginate 2%, sunflower vegetable oil 10%, prebiotic 2% and water up to 100 mL. All these % are in *w*/*v*. The obtained emulsion was mixed with a homogenizer (Velp Scientifica OV5 Homogenizer, Velp Scientifica SRL, Usmate Velate, Italy) at 10,000 U for 15 min, then autoclaved. After cooling, the emulsion was mixed again with the homogenizer at 10,000 U/min on ice for 30 min, in sterile conditions. The culture obtained from the third reactivation was centrifuged at 3000× *g* for 10 min at 4 °C ± 1. From here, 10 mL of pellet were taken and incorporated into the emulsion and mixed on a magnetic plate at 150 rpm for 15 min. The emulsion was injected using a peristaltic pump (Masterflex Peristaltic Pump 77200-62Easy-Load Pump Head, Cole Parmer, Vernon Hills, IL, USA) at 3.2 and 6.9 rpm/mL (depending on the type of emulsion) through a needle with a diameter of 0.3 mm. The suspension drip into glasses with sterile solution of calcium chloride (2%) from a height of 5 cm. Once the drops reached the calcium chloride solution, they immediately formed micrometric gel spheres. The obtained microcapsules were left to stay for 30 min for curing, then washed with sterile distilled water and recovered using a vacuum pump (EZ-Stream^®^ vacuum filtration pump, Merck, Vernon Hills, IL, USA).

### 2.4. Cell Viability

To determine the number of living cells from the emulsion, serial dilutions in sterile distilled water were performed. Then, 1 mL of the final dilution was inoculated in triplicate in Petri dishes with freshly prepared MRS agar and pre-cooled at 37–40 °C. The Petri dishes were incubated at 37 °C for 48–72 h under anaerobic conditions using anaerobic jars. The results obtained were reported in CFU/mL.

#### 2.4.1. Cell Viability in Microcapsules

The number of viable cells encapsulated in the matrix was determined as the following: 0.2 g of fresh microcapsules was dissolved in 9.8 mL of sterile 1% sodium citrate solution at pH 6.0. The tubes were gently shaken for 10 to 12 min at room temperature. Once the cells were released from the microcapsules, serial dilutions were made, in triplicate, with sterile distilled water. From the last dilution, 1 mL was inoculated in triplicate in Petri dishes with MRS agar. These were then incubated under anaerobic conditions at 37 °C for 72 h. The results obtained were expressed in CFU/g of microcapsules.

#### 2.4.2. Efficiency of the Microencapsulation Process (E%) and Viability Rate (R%)

The efficiency of microencapsulation represents the survival rate of micro-organisms during the microencapsulation process and calculated according to the following Equation (1) [25]:

E% = N_1_ ÷ N_0_ × 100 (1)

where N_1_ represents the number of viable cells (log_10_ CFU g^−1^) released from the microcapsules and N_0_ represents the number of viable cells (log_10_ CFU g^−1^) from the cell concentrate (emulsion) used for microencapsulation.

The cell viability rate (from microcapsules) as a result of the microencapsulation process (R%) was calculated according to Formula (2) used by Rather et al., 2017; Chávarri et al., 2010:

R% = log_10_N_1_ ÷ log_10_N_0_ × 100 (2)

where log_10_N_0_ represents the number of viable cells trapped in the capsule and log_10_N_1_ represents the amount of free viable cells added to the emulsion. The result of the equation is expressed as the number of CFU/mL.

#### 2.4.3. Viability of Encapsulated Bacteria after Enapsulation and during Storage

After being recovered from calcium chloride and washed, the microcapsules were stored in sterile Petri dishes at 4 °C. Depending on the type of encapsulation matrix, they were stored and checked for cell viability for periods of 30 to 45 days. Testing began with time 0 (T_0_), immediately after the encapsulation process, then at 7-day intervals (T_7_, T_14_, T_21_, T_30_). The same protocol described above was used. After the capsule dissolution, serial dilutions were performed and then inoculated into MRS agar. Afterwards, the petri dishes were incubated at 37 °C for 48–72 h under anaerobic conditions. Both dilutions and inoculations were performed in triplicate and the results obtained were expressed as log_10_ CFU/g^−1^ microcapsules.

#### 2.4.4. Cell Viability in Simulated Gastro-Intestinal Juices

Digestive juices were reconstituted according to the recipes described by Ashwar et al.,2018 and De Prisco et al., 2016 and adapted according to the protocol recommended by Minekus 2014.

##### Survival of Microencapsulated Cells in Simulated Gastric Juice

In a sterile saline solution (sodium chloride (NaCl) 0.9 g, purified water 100 mL), 3 g/L pepsin (Pepsin, for biochemistry, powder, ACROS Organics™, Activity: 0.5 U/mg) was added. The pH of the solution obtained was adjusted by 1.0 mol/L HCl to a value of 3.0 using the pH meter in sterility conditions [9,26,27,28]. For the preparation of gastric juice, distilled water heated at 38–39 °C was used. The testing of the viability of Lactobacillus strains for an hour in the simulated gastric juice was performed for those from the fresh microcapsules as well as for the strains which were stored for various periods of time at 4 °C. The same method described above was used to determine the cell viability of microcapsules in juices (Section 2.4.1). After the elapsed time, the gastric juice was aspirated and intestinal juice was added over the capsules. To test cell viability in gastric juice, after it was aspirated, the capsules were dissolved in sodium citrate to release the cells. The method described above was followed to verify the number of viable cells. The number of cells encapsulated in gastric juice was determined as CFU/g^−1^.

##### Survival of Microencapsulated Cells in Intestinal Juice

The simulated intestinal juice, enzyme free, was prepared by dissolving bile salts (Bile extract porcine, Product Number B8631, Sigma-Aldrich, Nurnberg, Germany) in intestinal solution (0.65% NaCl, 0.0835% KCl, 0.022% CaCl_2_ and 0.1386% NaHCO_3_) to result in a final concentration of 0.3% and pH 7.5 at 37 °C (Rather et al., 2017). To test cell viability, 0.2 g of microcapsules were suspended in 9.8 mL of intestinal juice and then incubated in a thermostat at 37 °C for a period of 60, 120, 180 and 240 min. The tubes were gently shaken at short intervals. The microcapsules tested in the simulated intestinal juice were:

(a) fresh microcapsules;
(b) fresh microcapsules that have been subjected to gastric acidity for 1 h. After that, the gastric juice was aspirated and intestinal juice was added;
(c) microcapsules that have been subjected to storage time actions at 4 °C;
(d) microcapsules that have been subjected to both the action of storage time at 4 °C and gastric juice.

After each established incubation time, the survival of the microencapsulated cells was immediately controlled by inoculation into MRS agar according to the protocol described above. Each determination was made in triplicate and the results obtained were expressed as CFU/g^−1^.

### 2.5. Characterization of Capsules

Given the dimensions of the micrometer, the morphological characterization and the dimensions of the microcapsules were observed, photographed and measured using optical and electron microscopy. Capsule surface morphology was characterized using a scanning electron microscope (SEM, VEGA II LMU–Tescan, Czech Republic)). A number of 30, 40, 55 microcapsules wet and dry were randomly analyzed depending on the type of matrix and the experiment. Drying of the microcapsules was performed by incubation in a Petri dish in a thermostat for one hour at 37 °C.

Several types of microcapsules were examined using an optical microscope as follows:

- microcapsules subjected to acidity for one hour in gastric juice;
- microcapsules subjected to intestinal juice for one hour;
- microcapsules initially subjected to one hour in gastric juice and then for one hour in intestinal juice.

### 2.6. Statistical Analysis

All experiments and analyses were performed in triplicate. Results were expressed as mean ± standard deviation. Statistical differences between groups were determined using the single variance analysis test (ANOVA), followed by the Tukey HSD, Scheffé, Bonferroni and Holm tests. The latter are multiple comparison tests that identify which of the pairs under analysis are significantly different from each other. The values considered statistically significant were those of 5% (*p* < 0.05).

## 3. Results and Discussion

The main objective of the study represents the determination of physicochemical properties of microcapsules that have in their matrix different sources of carbon and sodium alginate. This study also offers data about the effect of prebiotics in the sodium alginate microencapsulation process, information regarding the efficiency of the encapsulation process, as well as data about the biomass viability during storage for 30 days at 4 °C and during gastrointestinal simulation tests after encapsulation and storage.

### 3.1. Evaluation of the Efficiency of Prebiotic Activity

The replacement of glucose from the original MRS culture medium with prebiotics, promoted the growth of the three strains of lactobacilli, from the mixed culture under study, during the incubation at 37 °C (Table 1). During the 10 h of in vitro cell biomass growth monitoring at 37 °C, it was observed that in the culture media used as control, MRS WG and MRS G, the lactobacilli had a slight increase in the first 4 h. It is assumed that due to an increased metabolic energy requirement, in the case of MRS WG and MRS G, the nutritional reserves were limited and therefore the number of viable cells began to decrease starting with the 6th hour of monitoring. From the obtained data, it is observed that the cellular biomass increased more (*p* < 0.05) in the presence of inulin compared to the other substrates. Following the statistical analysis, it was found that at time 0 there are no significant differences (*p* > 0.05) between the increases of cellular biomass on different media. After two hours, the increase in cellular biomass begins to show significant differences between most media except between MRS WG and MRS STH where *p* > 0.05. The same after 6 h, except that there is no significant difference between the increase of cellular biomass on the MRS OLI and MRS STH medium (*p* > 0.05). The same situation is found after 10 h of cell growth monitoring, if between all other culture media, a significant difference was observed (<0.05), between MRS OLI and MRS STH, it does not exist (*p* > 0.05).

The fact that the inulin medium was favored for the increase of cellular biomass in detriment of starch medium can be explained by the complex chemical structure of starch compared to that of inulin and oligofructose. The starch chemical bonds may have cleaved more difficult as a result of the metabolic process of microorganisms. It is also possible that the autoclaving process of the media to have an impact on the metabolism of carbon sources. If we return to the chemical structure of the prebiotics studied, it is known that starch is a mixture of two polysaccharides, namely amylose and amylopectin. Potato amylopectin groups are relatively small and consist of 5–10 short chains. Amylopectin is the main component of starch, and in potato starch, it normally constitutes 70–80% off weight, regardless of the granules size. At the same time, the starch macromolecule consists of a large number of relatively short chains with a medium degree of polymerization (DP) of 21–28 residues [29,30,31]. As for inulin and oligofructose, they are composed of several fructose-fructose bonds which constitute the majority of glycosidic bonds. Inulin consists mainly of beta (2–1) fructosyl-fructose bonds and a glucose terminal unit. Inulin has a degree of polymerization (DP) of 10 to 20 (medium DP 6). Partial enzymatic hydrolysis of inulin results in oligofructose (DP 2–10; medium DP 4), which is a short-chain fructan [32].

The increase of the cellular biomass on the culture media with different types of carbon sources (INU, OLI, STH) showed the ability of lactobacilli strains to decompose the prebiotics studied. It seems that, as mentioned above, the chemical structure has an important role in the process of carbon sources metabolism by lactobacilli and the speed with which it occurs. Some lactobacilli species can use inulin in two stages, first to degrade it into fructose and then to use fructose molecules inside the cell [33]. As oligofructose is easier to metabolize due to its chemical structure, the preference of strains for this type of MSR OLI culture medium is observed. In the first 4 h of monitoring, the growth of cellular biomass on MRS INU was slower.

However, after the hydrolysis of inulin in oligofructose chains, a statistically significant increase was observed after 10 h of monitoring. Inulinase, hydrolysis enzymes, produced by probiotic strains act specifically on the β-2,1 bonds of inulin to produce fructose or FOS fructooligosaccharides [14,34]. Additionally, for MRS STH, a differentiation of cellular biomass growth (*p* < 0.05) is observed after 10 h of incubation compared to MRS INU. Amylolytic degradation of amyloses by α- and β-amylase and amyloglucosidase leads to glucose. Hydrolysis of amylopectin requires amylopululanase to cleave α- branch points (1 → 6); hydrolysis of amylopectin further produces α d-Glu-α- (1-6) -d-Glu (isomaltose) and oligosaccharides with mixed α- (1- → 4) and α- (1- → 6) bonds [35]. According to a study by Humblot et al. [36], who cultivated *L. plantarum A6* on MRS STH, simple carbohydrate consumption occurs in the first 3 h, followed by maltodextrins with 3 to 7 glucose units.

**Figure 1:**
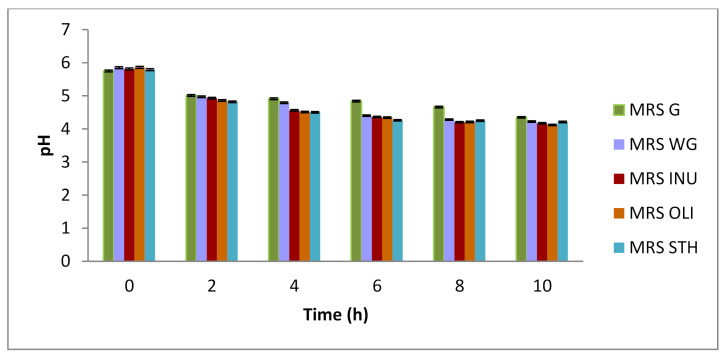
Changing of pH as a consequence of increasing cellular biomass in modified culture media. Each bar presents the mean of three replicates ± standard deviation (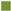 MRS G-culture medium made according to the original recipe, glucose culture medium, 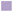 MRS WG-culture medium without carbon source, glucose-free culture medium, 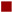 MRS INU-culture medium with inulin as a carbon source, 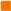 MRS OLI-culture medium with oligofructose as a carbon source, oligofructose culture medium, 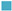 MRS STH-culture medium with potato starch as a carbon source, starch culture medium).

The effectiveness of prebiotic activity was also observed by monitoring the evolution of the acidity of the culture medium *(*Figure 1) for each type of prebiotic. As for the evolution of pH, the results showed that after 10 h of incubation, that with the increase of the cellular concentration, the pH decreases with each reading of the interval. Thus, the pH decreased from 6.2 to 4.17 ± 0.012 SD for inulin, to 4.12 ± 0.01 SD for OLI and 4.21 ± 0.01 SD for STH. The increase in the acidity of the media occurred as a result of the consumption of the carbon source and, probably, its transformation into organic acids [37]. This is due to the fact that homofermenting bacteria ferment sugars mainly in lactic acid and heterofermenting bacteria in addition to lactic acid also produce other products such as: acetic acid, ethanol, CO_2_ and formic acid, diacetyl, acetoin and 2,3-butanediol from citrate, bioactive peptides from amino acid catabolism and exopolysaccharides from carbohydrates [38].

### 3.2. Characterization of Microcapsules

The characterization of the microcapsule morphology was performed using optical and scanning electron microscopy. Their size can be influenced by factors such as: alginate concentration, calcium chloride concentration, needle diameter, pump pressure, distance between needle and calcium chloride solution, stirring speed, water-oil ratio [25,39]. Analyzing the morphology of the microcapsules with the help of the optical microscope, different shapes were observed: oval, ellipsoidal, drop, but the most abundant were the round ones. It has also been observed that the vast majority do not adhere to each other and the fact that after drying in an oven at 37 °C, the form was preserved.

**Figure 2:**
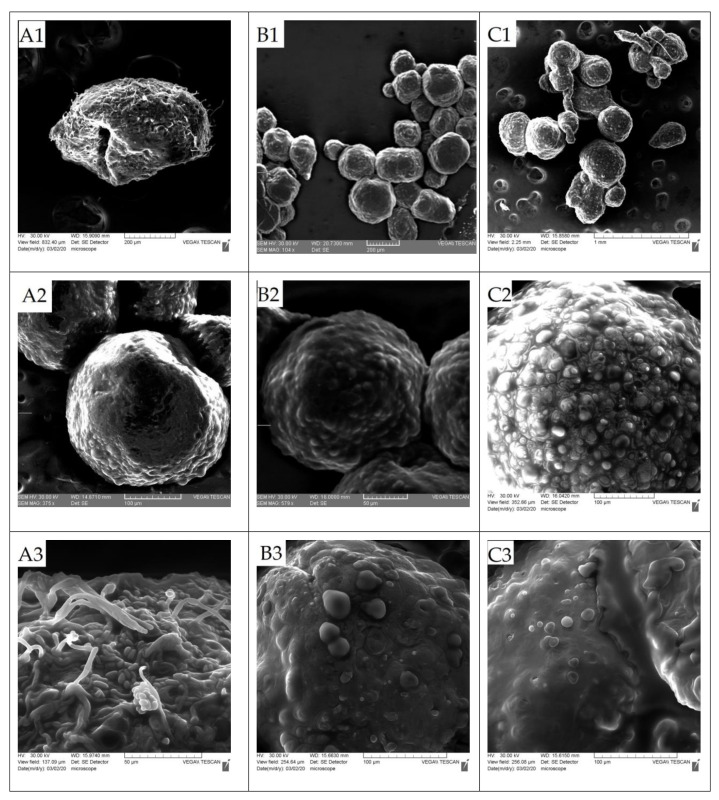

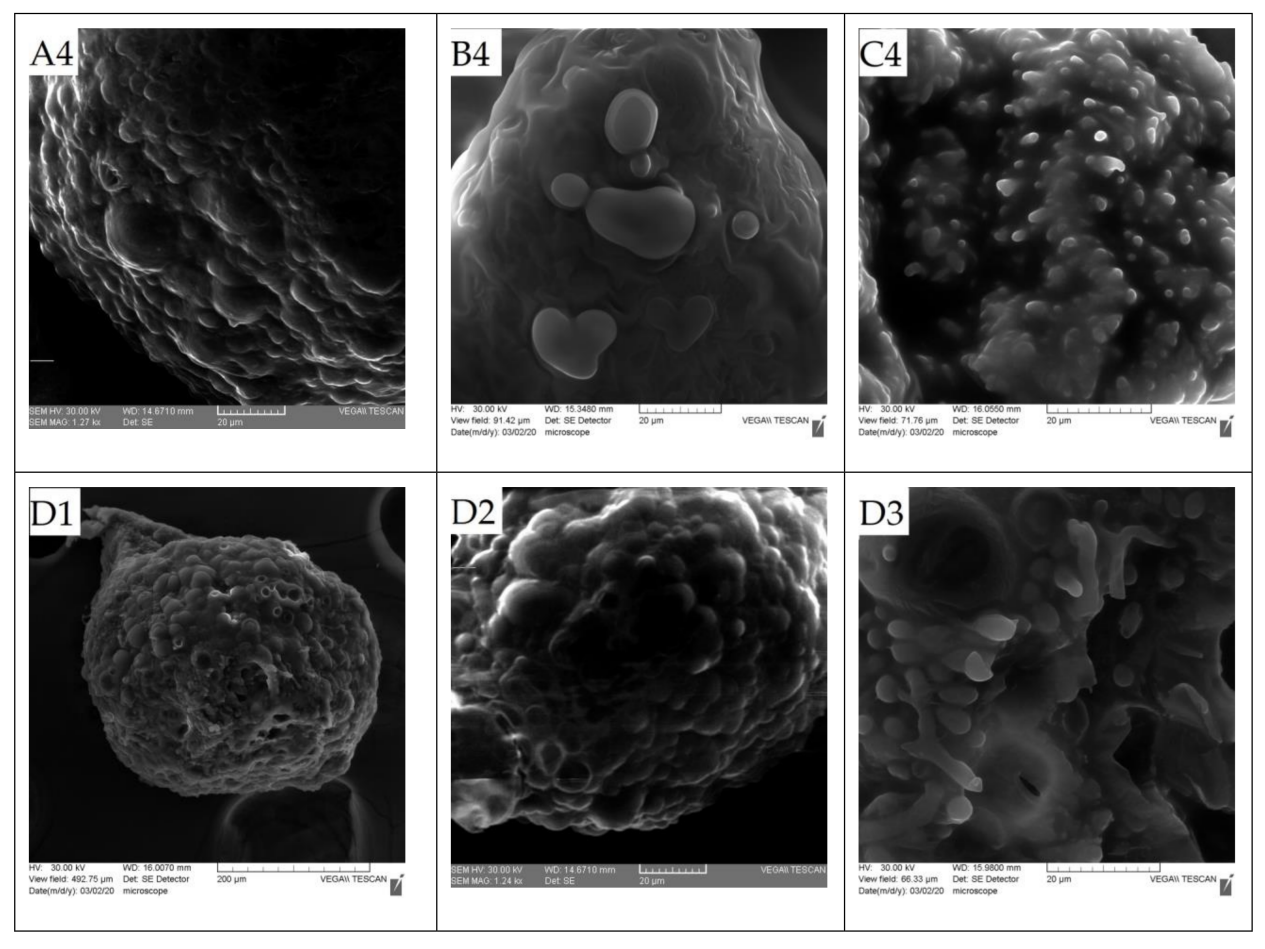
Capsule morphology—general image and wall image—prepared with different probiotics (**A**)–oligofructose, (**B**)–starch, (**C**)–inulin, (**D**)–glucose) captured with scanning electron microscope. (**A1**,**A2**,**B1**,**B2**,**C1**,**C2**) and (**D1**)—microcapsule image, (**A3**,**A4**,**B3**,**B4**,**C3**,**C4**) and (**D2**,**D3**)—microcapsule wall.

Following the analysis of SEM-scanning electron microscopy, in Figure 2, it was found that: the microcapsules were compact and continuous, spherical, but with an irregular surface. Fareez et al. [40] considered that this uneven surface is due to a high concentration of polymer, in this case, it was only 2%. The absence of *Lactobacillus*-free cells on the surface of the capsules was also noted, which indicates that the encapsulation process is efficient from this point of view.

It has been observed that the materials chosen for the encapsulation matrix offer different aspects of the microcapsule surfaces. It is observed that the surface of the microcapsules that have glucose in their matrix is in some places (here and there) porous. Instead, in the other microcapsules, the surface is with small unevenness/small bumps but without visible pores.

#### 3.2.1. Evaluation of the Average Diameter and Size Distribution of the Microparticles

**Figure 3:**
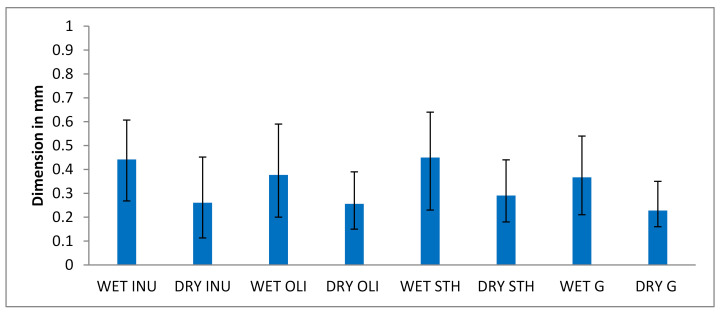
The dimensions of the wet and dry microcapsules in mm. Each bar presents the mean of three replicates. OLI—Microcapsules with oligofructose, STH—Microcapsules with starch, G—Microcapsules with glucose, INU—Microcapsules with inulin, the size is represented by the diameter of the microcapsule.

Factors such as stirring speed, surfactant concentration or water-oil ratio (*v*/*v*) may influence the distribution and size of the microcapsules. The microparticles showed micrometric dimensions (μm) as it can be seen in Figure 3 and they are accepted as optimal for food applications. The size of the particles in the micron range can provide a smooth texture to the food, while particles in millimeter sizes can provide a grainy texture [41]. Moreover, particles larger than 100 μm are needed to provide protection to probiotic cultures in simulated gastrointestinal conditions [42,43]. We assume that in this case, the intake of edible oil, the concentration and chemical structure of the chosen prebiotics, the concentration of alginate but also the speed and distance from which calcium chloride was dripped, played an important role in obtaining these dimensions. At the same time, the distance of 5 cm to the calcium chloride allowed that, if it is observed during the technological process, the formation of large drops is easily removed before dripping into the calcium chloride. Which is why we assume it would be responsible for the insignificant differences (*p* > 0.05) between Wet oli and Wet G and between Wet INU and Wet STH. Thus, 74 g of microcapsules were obtained from 100 mL of emulsion. The optimal composition of the emulsion and the size of the microcapsules were obtained after numerous trials whose data are not published in the present study. Starting from the concentrations of different ingredients, the dimensions and diameters of the needles to the distance and speed of dripping and checking the viability in simulated digestive juices. It was observed that the size of the microcapsules decreases with their drying in the incubator at 37 degrees Celsius for one hour. Since the data from the consulted literature do not provide, as far as we know, such treatments to the microcapsules obtained by this encapsulation technique, in order to be able to compare the microcapsules obtained, the study continued to focus on wet microcapsules. Especially since the dimensions, according to the literature, fall within the requirements of food applications. Following the statistical analysis of the dimensions of wet and dry microcapsules, it was observed that between most of them, there are significant differences with *p* < 0.01 (Wet OLI vs.: Dry OLI, Dry STH, Wet INU dry G, wet STH and Dry INU; Dry OLI vs.: Wet STH, Wet G, Wet INU; wet STH vs.: Dry STH, Wet G, dry G AND Dry INU; Dry STH vs.: Wet G, dry G, Wet INU; Wet G vs.: dry G, Wet INU and Dry INU; Wet INU vs. Dry INU), for *p* < 0.05: Wet OLI vs. Wet STH. Insignificant difference (*p* > 0.05) was observed between Wet OLI vs. Wet G, Wet STH vs. Wet INU, but in general, it was present between most dry microcapsules: Dry OLI vs. Dry STH, Dry OLI vs. Dry G and Dry INU, Dry STH vs. Dry INU and Dry G vs. Dry INU.

Compared with other studies using the same encapsulation technique and the same material for the microcapsule matrix, the size obtained in the present study was found to be smaller (<1 mm). For example, other researchers such as: Mokarram et al. [44], Hansen et al. [45] obtained microcapsules with dimensions larger than 1 mm. In other studies aimed at microcapsulation by extrusion, Lenton et al. [46] obtained an average size for microcapsules of 2.9 mm; Muthukumarasamy et al. [47] obtained an average size of 2.37 mm with a similar needle (G21). Using the same type of needle, Valero-Cases et al. [8] obtained an average value of 1.86 mm.

**Figure 4:**
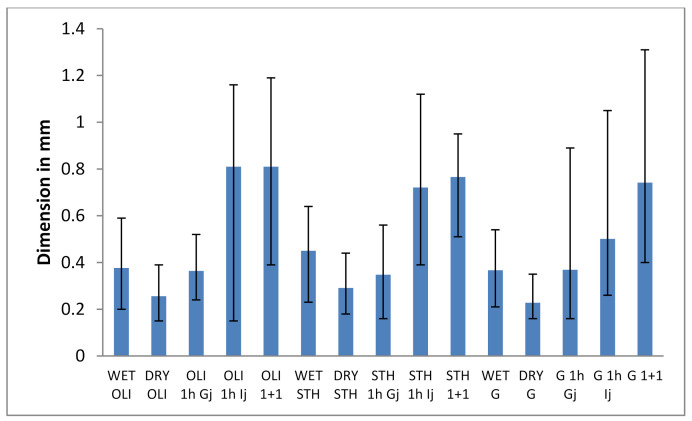
The effect of simulated gastrointestinal juices on the size of the microcapsules. Each bar presents the mean of three replicates. OLI—Microcapsules with oligofructose, STH—Microcapsules with starch, G—Microcapsules with glucose, INU—Microcapsules with inulin, 1 h Gj—Microcapsules that stood for one hour in simulated gastric juice at 37 °C, 1 h Ij—Microcapsules that stood for hour in simulated intestinal juice at 37 °C, 1 + 1—Microcapsules that stayed one hour in gastric juice then one hour in intestinal juice at 37 °C.

The action of intestinal juices brought changes in the size of the microcapsules. Therefore, in the gastric juice, under an optical microscope, a slight reduction in size was observed, while in the intestinal juice, the size was doubled and tripled in some microcapsules (Figure 4).

According to studies on alginate and its compounds performed by Smidsrod and Draget [48], these changes in microcapsule size are due to the junction areas of the calcium ion gel network. Initially, following the microencapsulation process, when the emulsion droplets came in contact with calcium ions, almost instantly, calcium alginate was formed on the surface of the sodium alginate droplets and maintained the shape of the droplets.

The formation of calcium alginate gel shows the phenomenon of syneresis or water loss associated with an increase in polymer concentration [49]. This leads to the fact that the polyguluronic sequences of alginate together with calcium ions produces the junction areas of the gel network. As the junction areas form the surface of the alginate gel, the change in the volume of the microcapsules must correspond to the number of junction areas.

Capsule compression can also be explained by the partial expulsion of inland water due to the addition of probiotic cells in emulsion and its extrusion by needle in calcium chloride with the formation of the gel layer [25,50,51]. The size of the microcapsules was reduced in the simulated gastric juice regardless of the prebiotic material used. This suggests that some of the water content of the microcapsules is released. The decrease is supposed to be a result of the reduction in electrostatic rejection between the chains due to protonation of the free carboxyl groups on the alginate. Moreover, due to the dissociation of calcium ions at low pH, an acid gel can form in which COO groups become protonated, allowing alginate chains to approach and form hydrogen bonds [52].

All microcapsules shrank under gastric conditions, probably due to reduced electrostatic repulsion forces at protonation at low pH with the dissociation of calcium ions from the gel and the formation of an acid gel. Once placed in the intestinal solution, all types of microcapsules began to swell to varying degrees. Under these conditions, the structure of the gel is weakened under conditions of high salt concentrations. It has been observed that some microcapsules disintegrate after prolonged periods in intestinal conditions. This is probably due to the increase of the electrostatic repulsive forces at a pH above pKa of the uronic acid groups on the alginate. These results reflect changes in pH and conditions of increased salt concentrations during the various stages of simulated digestion [49]. It was observed that at the end of the gastric phase, the microcapsules had a much closer and denser gel network than the one observed at the end of the intestinal phase which was more open and porous in nature. This is due to the pH sensitive changes of alginate, which leads to shrinkage and swelling in gastrointestinal conditions. The porosity of the microcapsule appears to be increasing, and towards the end of the intestinal phase, they start to disintegrate. This leads to the fact that the microcapsules obtained are an attractive option as devices for the controlled release of probiotics in this part of the gastrointestinal tract.

#### 3.2.2. Number of Viable Cells. The Efficiency of Microencapsulation

Maintaining cell viability during and after the encapsulation process is a very important factor regardless the type of encapsulation. The data obtained for the cell viability before and after the microencapsulation process are presented in Table 2. Analyzing the statistical analysis for each one, the results showed that in terms of cell viability in emulsions, there are no statistical differences in significance (*p* > 0.05) between: the emulsion containing G and the one with STH, the emulsion containing G and the one with OLI, and between the one containing OLI and the one with STH. The rest show statistically significant differences, *p* < 0.01. Regarding the cellular viability of microcapsules after encapsulation, statistically significant differences (*p* < 0.01) were obtained between all types of microcapsules, except for only the cell viability of microcapsules with G and microcapsules with STH (*p* > 0.05). At the same time, there were insignificant differences in microencapsulation yield for all 4 types of prebiotics (*p* > 0.05).

The percentage yields obtained after microencapsulation show that there is variability in the ability of lactobacilli strains to bind to the encapsulation matrix [27]. Some researchers [27,53] have stated that the yield of microencapsulation can be influenced by the material used for the matrix wall, the cell load in the emulsion, the chosen microencapsulation technique, the capsule size and the hardening time in calcium chloride. Other authors obtained an encapsulation efficiency of 88.19% [54] using the technique of extruding an emulsion of sodium alginate, inulin and *L. acidophilus*.

Raddatz et al. [55] used as an internal emulsification/gelling encapsulation technique, obtaining an encapsulation efficiency between 82.65% and 91.24%. In some studies, using a similar technique for encapsulating *Bifidobacterium bifidum F-35* [56], lower values were obtained, between 43% and 50%. By [9], who used the same technique as in the present study to encapsulate *L. plantarum NCDC201* and *L. casei NCDC297*, using a thicker needle (G21-which led to higher microcapsules) and obtained a lower yield than in the present study, namely 72.48% and 62.54%. In this study, it can be observed that regardless of the concentrations used, optimal encapsulation yields were obtained for all but with insignificant differences between them (*p* > 0.05).

### 3.3. Viability of Encapsulated Probiotics during Storage

In order to monitor the stability of the microcapsules under refrigeration conditions at 4 °C, they were stored in Petri dishes. The cell viability of microcapsules stored in the refrigerator at 4 °C is shown in Table 3. The values obtained for cell viability from the same storage time (e.g., 0 days = T_0_) and the same type of capsules were statistically analyzed and it was found that there are significant differences (*p* < 0.01), except for only the cell viability between G and STH microcapsules (*p* > 0.05). At the same time, all possible pairs/contrasts from the same storage moment were evaluated statistically INU T_0_-G T_0_, INU T_0_-STH T_0_; INU T_0_-OLI T_0_; OLI T_0_-STH T_0_; OLI T_0_-G T_0_; STH T_0_-G T_0_, followed by T_7_, T_14_, T_21_, T_30_). After 14 and 28 days of storage, respectively, the statistical analysis made between the probiotic cell viability of the different types of microcapsules indicates statistically significant differences between all without exception. Overall, the microcapsules that have as carbon source glucose, have the lowest cell viability at each moment of storage. These facts are well highlighted in Table 3 which represents the survival rate during storage.

It was found that the survival rate of lactic strains is influenced by the type of prebiotic used as a compositional part of the microcapsule matrix. This resulted from the statistical analysis where significant differences were recorded (*p* < 0.05) and can be seen in Table 3. The highest survival rate at T_14_ was obtained for INU: 72%, while for STH it was 66.43%, OLI: 62.69% and G: 55.76%. At T_30_ the highest survival rate was registered for STH at 63.98% compared to 62% INU, 58.23% OLI and 48.17% G. However, referring to the number of cfu/g, INU registries a value of 9 log10 cfu/g while STH registries a value of 8.98 log10 cfu/g. After 30 days of storage OLI had a cell viability of 8.33 log10 cfu/g which is very close to the other two types of microcapsules (Table 4). The data obtained show that the presence of a source of carbon in the matrix of the microcapsule provides the possibility of cellular metabolic activity at 4 °C.

The decrease in cell viability during the storage period is not due to the encapsulation process. Some of the main causes for the decrease in survival rate could be time, consumption of the nutrient substrate and the presence of compounds resulting from the metabolic process (acids, bacteriocins). Another factor involved in decreasing cell viability would be the increased water activity but also the presence of residual water in micro-capsules. At each time of analysis, the plates in which the microcapsules were stored were permanently open, which would have increased the degree of humidity [45]. Humidity is also known to have a negative effect on cell viability [57]. Analyzing other studies that used the same encapsulation method, but different strains (*L. gasseri* and *Bifidobacterium bifidum*), it was found that a decrease in viability in the first 11 days of 4.11 cfu log/g was reported; and after 14 days, they did not observe survival [53].

### 3.4. Cell Viability in Simulated Gastrointestinal Conditions

In the literature, various models of digestion and various recipes of digestive juices are proposed. This diversity leads to the impossibility of comparing the results obtained with other research topics due to the fact that:

- a wide variety of enzymes used that differ in their activity and characterization.
- the enzymes used come either from different sources of animal origin such as rabbits, pigs, or even of human origin.
- differences in pH, mineral type, ionic strength, presence or absence of phospholipids, gastric lipase, pancreatin were found.
- the products are subjected to the action of digestive juices for different periods, and different durations of enzymatic activity can significantly change the results [26].

For this study, research reports were selected for comparison that follows in the most accurate lines the same recipes and the same parameters. In the microcapsules, it is necessary to have a sufficiently high probiotic load so that when it reaches the place of action in the intestine, it has an optimal number for an efficient colonization.

**Figure 5:**
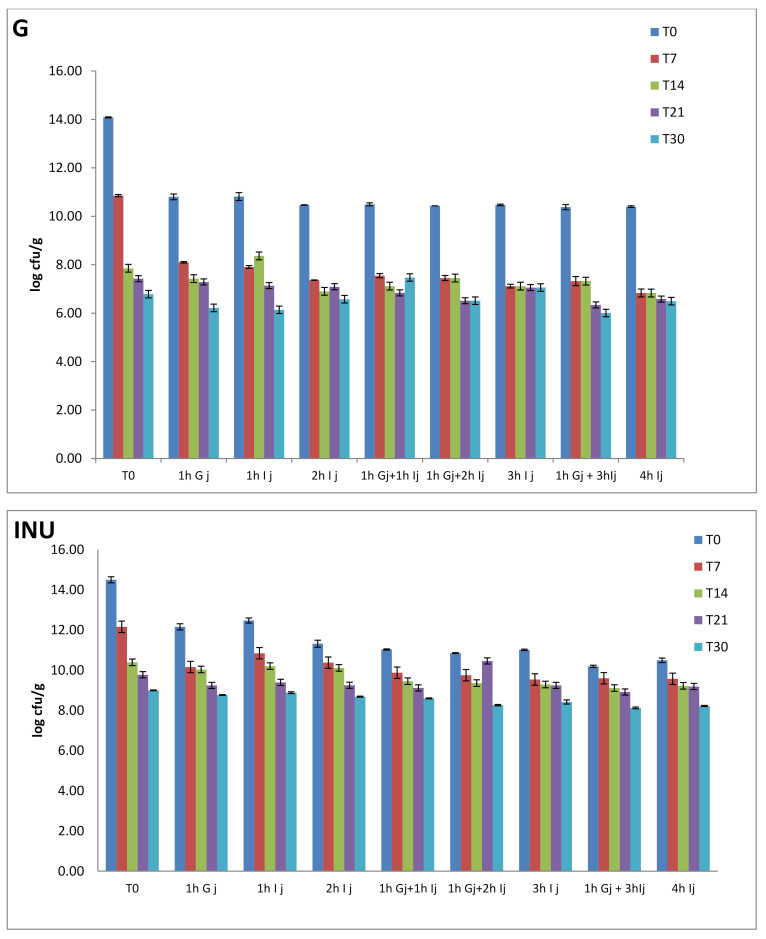

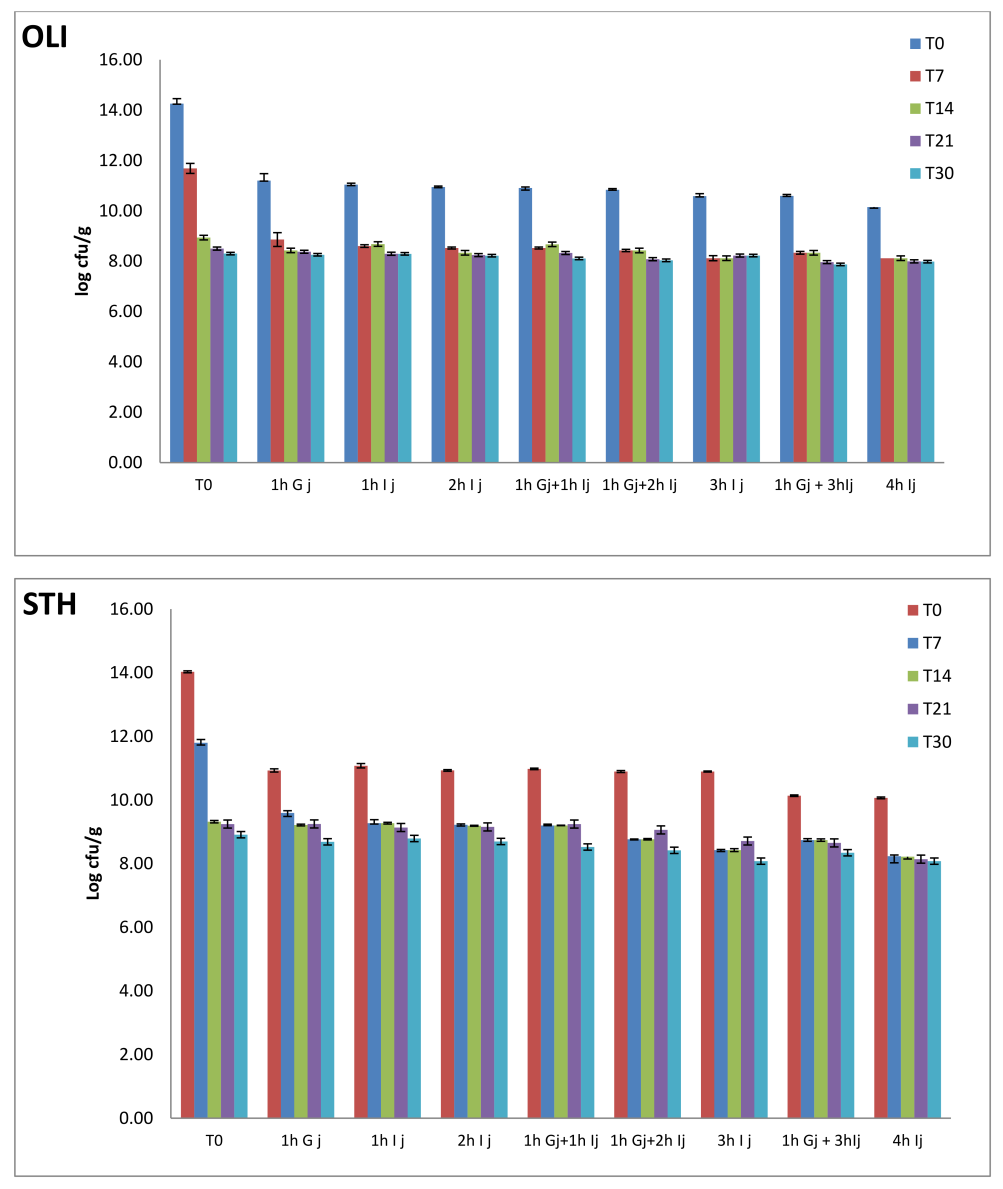
Cell viability in simulated gastrointestinal juices for microcapsules. 1 h Gj—viability in microcapsules that stayed for one hour in the simulated gastric juice at 37 °C, 1 h Ij—viability in microcapsules that stayed for one hour in the simulated intestinal juice at 37 °C, 1 h Gj + 1 h Ij (2 h Ij; 3H Ij)—viability in microcapsules that stayed one hour (two, respectively three hours) in the gastric juice then one hour in the intestinal juice at 37 °C. T0, T7, T14, T21, T30—storage moments (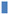
—T0, 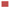
—T7, 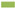
—T14, 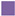
—T21, 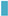
—T30).

After 30 days, the lowest survival rate of *Lactobacillus* cells in juices was observed in the microcapsules that have glucose as a carbon source. After 4 h in the intestinal juice, the survival rate is 46.11% while, after one hour under the action of gastric juice and then another 3 h in intestinal juice, the survival rate is 42.65% (Figure 5). This confirms those mentioned in the analysis of dimensions, namely that the action of gastric juice compresses the walls of the capsule while the intestinal juice leads to their dilation. Cell viability in simulated gastrointestinal juices from microcapsules after refrigeration for 30 days at 4 °C is shown in Figure 5.

Further, pH differences (from 3 to 7.5) led to a decrease in the viability rate by 3.47%. The difference is visible in all types of microcapsules. Cell viability is lower with the passage of capsules through both types of juices, compared to those that have been in intestinal juice for the same number of hours. If at time T_0_, the highest cell viability after the treatment at 1 h G j + 3 h Ij was found in the microcapsules with OLI (10.59 log10 cfu/g), at T_7_, the highest viability was found in those with INU (9.60 log10 cfu/g) followed by STH (8.74 log10 cfu/g). Viability for OLI decreased to 8.33 log10 cfu/g and remained constant at T_14_. The same stability was found at STH (8.74 log10 cfu/g), while for INU, the viability decreased to 9.11 log10 cfu/g. Cell survival in capsules seems to remain at about the same values for STH at T_21_ as the decrease compared to T_14_ was only 0.09 log10 cfu/g, but with a statistically significant difference (*p* < 0.05). INU decreased by 0.20 log10 cfu/g and OLI by 0.38 log10 cfu/g.

Even if at the end of the storage period, the viability of probiotic strains in the microcapsules subjected to treatment at 1 h Gj + 3 h Ij decreased compared to T_21_ by only 0.09 log10 cfu/g for OLI, the highest viability was found in STH. The values for OLI were 7.87 log10 cfu/g, INU-8.12 log10 cfu/g and STH-8.34 log10 cfu/g and G-6.01 log10 cfu/g. These values indicate that all carbon sources used to maintain high viability have achieved the objectives pursued. It was found that after 4 h of action of gastrointestinal juices, cell viability falls within the limits recommended by the FAO. However, in order to survive the passage through all the severe conditions of the digestive tract and to provide a beneficial action, the cell concentration may be increased during encapsulation relative to the carbon source, which would lead to an increased number of probiotic survivors at the end of incubation in simulated gastric conditions.

## 4. Conclusions

This study compared the influence of three carbon substrates to increase the biomass of a mixed probiotic culture of *Lactobacillus casei*, *L. plantarum* and *L. rhamnosus*. Carbohydrate polymers with a relatively low degree of polymerization were selected as they are considered the best encapsulation substrates [58]. The results showed that biomass production and substrate consumption increased significantly over 10 h for the culture medium supplemented with prebiotics. The three carbon sources tested led to an improvement in stem cultivation. Afterwards, probiotics viability and encapsulation degree were tested in the most favorable substrates. Studies have been performed on inulin, oligo-fructose and starch determining the most effective concentrations, encapsulation ratio and viability of the microencapsulated probiotics under simulated gastrointestinal conditions.

Microencapsulation with STH and INU improved the viability of Lactobacillus strains during the microencapsulation process, subsequent storage but also following the action of simulated gastrointestinal juices. These results showed that the strains of *L. casei*, *L. plantarum* and *L. rhamnosus* encapsulated with INU, OLI and STH that have in the composition of the matrix a concentration of 2% and biopolymer 2% prepared by extrusion technique, survived better than those with glucose.

Because the combination of symbiotic (lactobacilli strains with the three carbon sources) and biopolymer (sodium alginate) has improved the stability of encapsulated bacteria, this extrusion microencapsulation technology could be used to develop functional foods with probiotics.

## Figures and Tables

**Table 1:** Viable count (log10 cfu/mL) of probiotics for 10 h of monitoring at 37 °C on MRS medium culture.

Time (h)	MRS G	MRS WG	MRS INU	MRS OLI	MRS STH
0	8.51 ± 0.01 ^d A^	8.53 ± 0.03 ^c A^	8.48 ± 0.02 ^a A^	8.49 ± 0.01 ^a A^	8.46 ± 0.02 ^a A^
2	8.96 ± 0.01 ^e D^	8.56 ± 0.01 ^c A^	8.75 ± 0.02 ^b B^	8.81 ± 0.01 ^b C^	8.57 ± 0.02 ^b A^
4	9.01 ± 0.01 ^e B^	8.62 ± 0.02 ^c A^	9.06 ± 0.01 ^c B^	9.17 ± 0.01 ^c C^	8.58 ± 0.03 ^b A^
6	8.32 ± 0.04 ^c B^	7.70 ± 0.02 ^b A^	9.15 ± 0.03 ^c C^	9.49 ± 0.01 ^d D^	9.48 ± 0.04 ^d D^
8	7.84 ± 0.02 ^b A^	7.61 ± 0.06 ^b A^	9.51 ± 0.03 ^d B^	9.51 ± 0.04 ^d B^	9.32 ± 0.04 ^e B^
10	7.30 ± 0.02 ^a B^	3.60 ± 0.01 ^a A^	9.64 ± 0.02 ^e D^	9.52 ± 0.01 ^d C^	9.54 ± 0.01 ^d C^

MRS G culture medium made according to the original recipe, MRS WG-culture medium without carbon source, MRS INU-culture medium with inulin as a carbon source, MRS OLI-culture medium with oligofructose as a carbon source, MRS STH-culture medium with potato starch as a carbon source. Values are mean ± standard deviation of three replicates. Small lettered superscripts are used to differentiate values between rows, while capital lettered superscripts to differentiate values between columns.

**Table 2:** Cell viability before and after encapsulation. Encapsulation efficiency %.

	log10 cfu/mL	log10 cfu/g	%
	Cell Viability in Emulsion	Cell Viability in Microcapsules	Encapsulation Efficiency
INU	16.45 ± 0.09 ^b^	14.50 ± 0.04 ^c^	88.16 ± 0.13 ^c^
OLI	16.17 ± 0.05 ^a^	14.26 ± 0.03 ^b^	88.19 ± 0.09 ^c^
STH	16.12 ± 0.08 ^a^	14.03 ± 0.03 ^a^	87.02 ± 0.12 ^a^
G	16.09 ± 0.04 ^a^	14.09 ± 0.02 ^a^	87.54 ± 0.11 ^b^

OLI—Microcapsules with oligofructose, STH—Microcapsules with starch, G—Microcapsules with glucose, INU—Microcapsules with inulin. The different “a–d” indices in the same column denote differences from a statistical point of view (*p* < 0.05) between the same categories of results, according to the Tukey test. The values are the result of three determinations and are expressed in mean ± standard deviation.

**Table 3:** Impact of shelf life on the survival rate of probiotic strains. T = time in days, T0–T7, T0–T14, T0–T21, T0–T30—storage times between which the survival rate was calculated.

Time	Glucose	Oligofructose	Starch	Inulin
T0–T7	77.06 ± 0.14 ^d A^	81.96 ± 1.28 ^c B^	84.11 ± 0.31 ^c C^	83.86 ± 0.82 ^d B C^
T0–T14	55.76 ± 0.24 ^c A^	62.69 ± 0.18 ^b B^	66.43 ± 0.12 ^b D^	71.67 ± 0.42 ^c C^
T0–T21	52.71 ± 0.25 ^b A^	59.62 ± 0.19 ^a B^	66.19 ± 0.12 ^b D^	67.42 ± 0.28 ^b C^
T0–T30	48.17 ± 0.97 ^a A^	58.23 ± 0.11 ^a B^	63.82 ± 0.28 ^a D^	62.07 ± 0.20 ^a C^

Small lettered superscripts are used to differentiate values between rows, while capital lettered superscripts to differentiate values between columns.

**Table 4:** Viability of lactobacilli strains during the storage period.

Storage Period	G	OLI	STH	INU
T_0_	14.08 ± 0.02 ^e A^	14.25 ± 0.02 ^d B^	14.03 ± 0.03 ^d A^	14.50 ± 0.04 ^e C^
T_7_	10.85 ± 0.04 ^d A^	11.68 ± 0.20 ^c B^	11.80 ± 0.07 ^c B^	12.16 ± 0.15 ^d B^
T_14_	7.85 ± 0.05 ^c A^	8.94 ± 0.04 ^b B^	9.32 ± 0.04 ^b C^	10.39 ± 0.09 ^c D^
T_21_	7.42 ± 0.05 ^b A^	8.50 ± 0.04 ^a B^	9.24 ± 0.04 ^b C^	9.77 ± 0.07 ^b D^
T_30_	6.78 ± 0.15 ^a A^	8.30 ± 0.00 ^a B^	8.98 ± 0.06 ^a C^	9.00 ± 0.02 ^a C^

The values (log10 cfu/g) are the result of three determinations and are expressed in the mean ± standard deviation, T0, T7, T14, T21, T30—storage moments. OLI—Microcapsules with oligofructose, STH—Microcapsules with starch, G—Microcapsules with glucose, INU—Microcapsules with inulin. Small lettered superscripts are used to differentiate values between rows, while capital lettered superscripts to differentiate values between columns (*p* < 0.05).

## Data Availability

Not applicable.
